# Optical coherence tomography angiography characteristics of acute retinal arterial occlusion

**DOI:** 10.1186/s12886-019-1152-8

**Published:** 2019-07-10

**Authors:** Shuai Yang, Xiaoqiang Liu, Hui Li, Jing Xu, Fang Wang

**Affiliations:** Department of Ophthalmology, Shanghai Tenth People’s Hospital, Tongji University School of Medicine, Shanghai, 200072 China

**Keywords:** Optical coherence tomography angiography, Retinal arterial occlusion, Vessel density, Acircularity index, Foveal avascular zone

## Abstract

**Background:**

To characterize the vascular changes in eyes within the acute phase of retinal arterial occlusion (RAO) by optical coherence tomography angiography (OCT-A) imaging.

**Methods:**

This was a retrospective, observational study. Nineteen patients with RAO (symptom onset within 7 days) and 19 age and sex-matched normal control individuals were included. A comprehensive ophthalmic examination and OCT-A examination were conducted for all the patients.

**Results:**

The vessel density of the superficial capillary plexus (SCP), deep capillary plexus (DCP), and area with a width of 300 μm around the FAZ (FD-300) was significantly reduced in RAO patients compared with that in the fellow eyes and normal control eyes. The vessel density of the SCP of RAO fellow eyes was significantly lower than that of the normal control eyes (all *P* < 0.05). Though no difference was observed in the FAZ of RAO eyes compared with that of fellow eyes and normal control eyes, the acircularity index (AI) of the FAZ was significantly increased in RAO eyes (*P* < 0.05). Central macular thickness (CMT) was correlated with best-corrected visual acuity in central retinal arterial occlusion (CRAO) patients (*r* = 0.626, *P* = 0.024). In BRAO eyes, the vessel density of the RAO-affected hemifield was significantly reduced compared with that of the unaffected hemifield (*P* < 0.05). Radial peripapillary plexus (RPC) vessel density was reduced, accompanied by retinal nerve fiber layer (RNFL) thinning in 3 available CRAO patients.

**Conclusions:**

As a valuable noninvasive imaging tool, OCT-A provides deeper and more detailed vascular information that extends our understanding of the vasculature alterations in acute RAO.

**Electronic supplementary material:**

The online version of this article (10.1186/s12886-019-1152-8) contains supplementary material, which is available to authorized users.

## Background

Retinal arterial occlusion (RAO) is characterized by painless, dramatic, sudden vision loss as a result of partial or complete obstruction of the retinal artery. RAO can be classified as central retinal artery occlusion (CRAO) and branch retinal artery occlusion (BRAO) based on the site of the occlusion (central retinal artery and branch retinal artery, respectively). Though the incidence of RAO is rare, the prognosis of this emergency is usually poor (especially for CRAO) partially because that there are no proven effective treatments for RAO.

In the clinic, fluorescein angiography (FA) is applied to visualize retinal vessels and evaluate the vessel flow in RAO patients. However, capillary networks in different layers of the retina cannot be distinguished or quantified by FA. In addition, FA is an invasive examination; thus, its frequent application is limited.

Compared to FA, optical coherence tomography angiography (OCT-A) is an innovative noninvasive imaging technique that has been applied in recent years. OCT-A visualizes the retinal and choroid capillary networks and foveal avascular zone (FAZ) without any exogenous dye. Split spectrum amplitude decorrelation angiography (SSADA) associated OCT-A (Optovue, Inc., Fremont, CA) can produce three dimensional and en face imaging of retinal capillary networks. The quantitative data of the retinal vasculature, as well as the thickness of the retinal vasculature and foveal avascular zone area can be calculated automatically by AngioAnalytics software.

Due to its convenience and safety, OCT-A is currently widely used in diagnosing and evaluating various vascular-associated retinal or choroidal diseases, such as diabetic retinopathy, choroidal neovascularization, and retinal vein occlusion [1–3]. Although several case reports and studies reported the application of OCT-A in RAO, these publications are sporadic and lack quantitative analysis [4]. In this study, we aimed to quantify retinal vascular density, foveal avascular zone (FAZ) area and central foveal thickness by OCT-A in patients with acute RAO (onset within 7 days), and compare the parameters with those in their fellow eyes as well as in normal control eyes.

## Methods

### Study design and subjects

This retrospective study included CRAO or BRAO patients hospitalized in the Department of Ophthalmology, Shanghai Tenth People’s Hospital from Jan 2017 to Nov 2018. RAO patients who experienced symptoms within 7 days were eligible for this study. The exclusion criteria included giant cell arteritis and any previous or current eye diseases except for RAO. Nineteen age- and sex-matched healthy individuals were also recruited as normal controls. This study was approved by the ethics committee of Shanghai Tenth People’s Hospital, and was in compliance with the Declaration of Helsinki. All individuals included in this study gave their written approval and consent for conducting this study and publishing in academic journals.

### OCT-A examination and measurements

After admission, all patients received treatment that included oxygen inhalation, a decrease of intraocular pressure, eyeball massage, and intravenous treatment alprostadil, Shu-Xue-Tong (traditional Chinese medicine), and mecobalamin, with intent to improve micro-circulation and reduce hypoxia-induced retinal damage. All of the patients received a comprehensive ophthalmological examination including best-corrected visual acuity (BCVA, converted to LogMAR for statistical analysis), measurement of the intraocular pressure, slit lamp biomicroscopy, fundus examination and photography, FA (Spectralis, Heidelberg Engineering, Heidelberg, Germany), and OCT-A (spectral domain system RTVue-XR Avanti (Optovue Inc. Fremont, CA) within 24 h after admission. Patients with BCVA of counting fingers were arbitrarily assigned a logMAR value of 2.3; hand movements, 2.5; light perception, 2.7; and no light perception, 2.9.

OCT-A scanning was conducted under Angio Retina mode (3 × 3 mm). Detailed information on OCT angiography, including the mechanisms of SSADA, has been previously described [5]. The motion artefacts of eyes were decreased by eye tracking mode and were removed by motion correction technology. After acquisition of the image, the software automatically segmented the tissue into 4 layers, and three of these layers were used in the following measurements. The superficial retinal layer starts from the inner limiting membrane (with an offset of 0 μm) to the inner plexiform layer with an offset of − 9 μm. The deep retinal layer starts from inner plexiform layer with an offset of − 9 μm to the outer plexiform layer with an offset of 9 um. The choriocapillaris layer from Bruch’s membrane layer with an offset of − 9 μm to Bruch’s membrane layer with an offset of 31 μm. For some patients, OCT-A scanning conducted with optic disc mode was performed (4.5 × 4.5 mm). The cutoff value of the signal strength index was set at ≥40.

The vessel density in the superficial retinal layer (superficial capillary plexus, SCP) and deep retinal layer (deep capillary plexus, DCP) was quantified automatically by AngioVue Analytics, RTVue-XR version 2017.1.0.155 software.

The central macular thickness (CMT, defined as the average thickness in the central 1 mm^2^ area centred on the fovea), parafovea macula thickness, average thickness of a 3 × 3 retina tissue area, and average retinal thickness of the superior hemifield and inferior hemifield were also quantified automatically by AngioVue Analytics software. The FAZ of the full retina, acircularity index (AI) of FAZ, and vessel density of the full retina in a width of 300 μm around the FAZ (FD-300) were also obtained in the FAZ mode of the software. The vessel density of the radial peripapillary plexus (RPC), peripapillary or inside disc, and retinal nerve fiber layer (RNFL) thickness were obtained in the optic disc mode.

### Statistical analysis

The statistical analysis was performed with the GraphPad Prism 5 software. Fisher’s exact test was used to test the difference between qualitative variables. In RAO patients, the RAO eye was defined as the eye affected by RAO within 7 days. The fellow eye was defined as the other eye (without RAO) in RAO patients. In healthy control individuals, both eyes were included as normal control eyes.

Differences in vessel density, retinal thickness, FAZ, and AI between eyes with RAO and fellow eyes were tested by paired student’s t-test. Furthermore, the parameters of normal control eyes were regarded as references. The parameters of RAO eyes and fellow eyes were compared with those of normal control eyes separately (two-tailed student’s t test). The Spearman correlation coefficient was used to assess correlations. Values are presented as the means ± standard deviation (SD). A value of *P* < 0.05 was accepted as statistically significant.

## Results

### Patient characteristics

Nineteen eyes from 19 patients (age: 66.47 ± 13.57, 16 males and 3 females) diagnosed with RAO were eligible for this study. Thirteen patients were diagnosed with CRAO, and 6 were diagnosed with BRAO. Nineteen age- and gender-matched individuals without any ophthalmic disorders were also enrolled as normal controls.

### OCT-A findings in RAO patients

In all cases, OCT-A images showed a typical ischemic appearance in both SCP and DCP, deficient capillary network, and darker background as a result of ischemia. These changes were correlated with the ischemic changes in FA (Additional file 1: Figure S1).

**Fig. 1:**
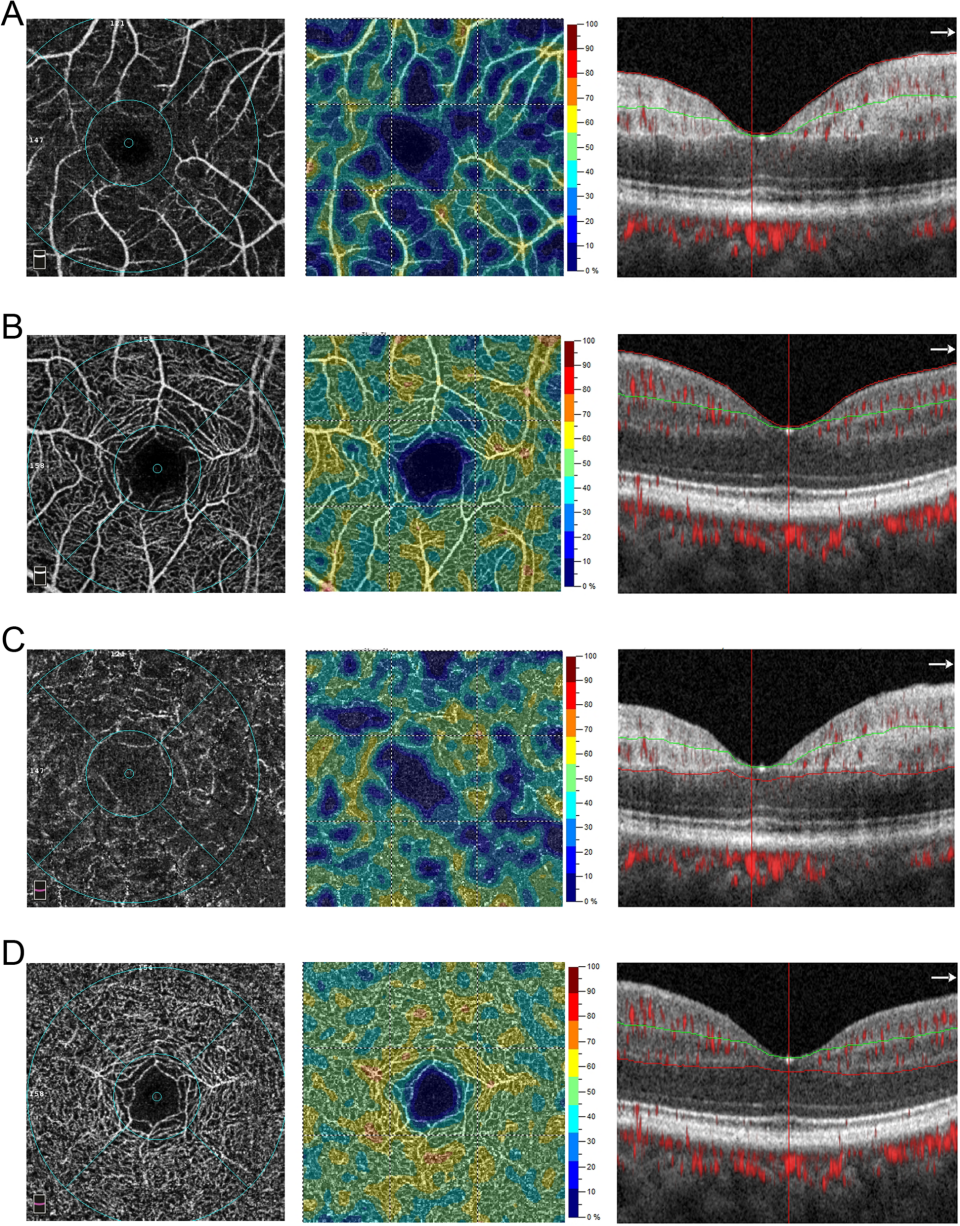
OCT-A image of a representative CRAO patient. **a**-**b** SCP, vessel density heat map of SCP, B-scan centered on the fovea, which shows the segmentation of the superficial retinal layer of the CRAO eye (**a**) and the fellow eye (**b**). **c**-**d** DCP, vessel density heat map of DCP, B-scan centered on the fovea, which shows the segmentation of the deep retinal layer of the CRAO eye (**c**) and the fellow eye (**d**)

**Fig. 2:**
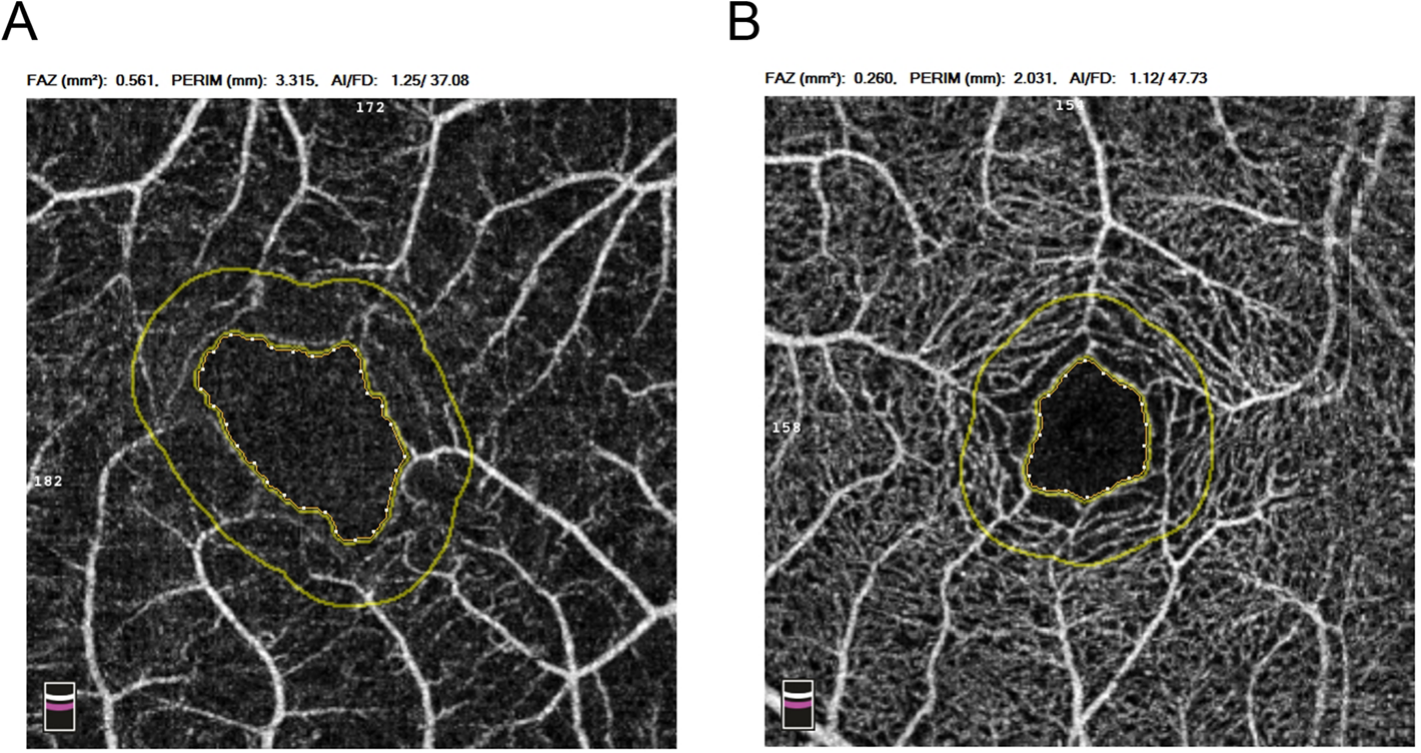
The FAZ mode of the OCT-A image of the same patient in Fig. 1. The border of FAZ, and 300 μm width outside the FAZ area (FD-300) were created automatically. **a** CRAO eye. **b** Fellow eye

Quantitatively, OCT-A imaging revealed a significant decrease in vessel density in SCP, DCP and FD-300 areas in RAO eyes compared with those in fellow eyes and normal control eyes (Figs. 1 and 2, Table 1). The CMT, parafoveal thickness, and overall thickness of the 3*3 mm cube were significantly increased in RAO eyes. The FAZ of RAO eyes showed no difference compared with that of fellow eyes or normal control eyes. Moreover, in RAO eyes, the AI is significantly higher than both the fellow eye and normal control eyes (Table 1, Fig. 2). In addition, between fellow eyes and normal control eyes, SCP showed a significant reduction, while other parameters showed no significant difference (Table 1).

**Table 1:** Vessel densities, FAZ area, AI of FAZ, and retinal thickness of RAO eyes compared with those of fellow eyes and normal control eyes

	RAO eyes (*n* = 19)	Fellow eyes (*n* = 19)	NC eyes (*n* = 38)	P1	P2	P3
VD in SCP	37.26 ± 5.85	41.98 ± 5.44	45.37 ± 6.28	**< 0.001**	**< 0.001**	**0.044**
VD in DCP	40.54 ± 4.96	45.88 ± 5.09	46.66 ± 5.51	**0.0011**	**< 0.001**	0.34
FAZ	0.38 ± 0.16	0.360 ± 0.13	0.36 ± 0.08	0.52	0.60	0.99
AI	1.17 ± 0.045	1.14 ± 0.04	1.15 ± 0.04	**0.015**	**0.0259**	0.61
VD in FD-300	42.58 ± 5.04	48.46 ± 3.27	50.20 ± 4.44	**< 0.001**	**< 0.001**	0.11
CMT	287.61 ± 55.48	249.42 ± 19.07	244.75 ± 17.78	**0.0093**	**< 0.001**	0.20
Parafoveal thickness	373 ± 51.12	317.68 ± 21.57	317.55 ± 21.38	**< 0.001**	**< 0.001**	0.62
Overall thickness	359.47 ± 43.91	307.63 ± 19.75	309.33 ± 20.68	**< 0.001**	**< 0.001**	0.88

VD: vessel density. A *P* value < 0.05 is considered statistical significance and are shown in boldface

P1: comparisons of RAO eyes vs fellow eyes (paired student’s t test)

P2: comparisons of RAO eyes vs NC eyes (unpaired student’s t test)

P3: comparisons of fellow eyes vs NC eyes (unpaired student’s t test)

Subgroup analysis was conducted by the type of RAO (CRAO or BRAO). Significant differences remained in AI; the vessel density of SCP, DCP, and FD-300; and retinal thickness parameters of CRAO eyes compared with those of fellow eyes (Table 2).

**Table 2:** Vessel densities, FAZ area, AI of FAZ, and retinal thickness of CRAO eyes compared with those of fellow eyes

	CRAO eyes(*n* = 13)	Fellow eyes (*n* = 13)	*P* value	BRAO eyes(*n* = 6)	Fellow eyes (*n* = 6)	*P* value
VD in SCP	36.98 ± 6.14	41.35 ± 6.20	**0.0041**	37.87 ± 5.66	43.35 ± 3.28	0.11
VD in DCP	39.85 ± 4.13	45.87 ± 5.90	**0.0060**	42.05 ± 4.96	45.90 ± 3.15	0.11
FAZ	0.38 ± 0.17	0.370 ± 0.147	0.833	0.38 ± 0.13	0.34 ± 6.20	0.41
AI	1.18 ± 0.052	1.140 ± 0.0374	**0.046**	1.17 ± 0.03	1.13 ± 0.32	**0.0029**
VD in FD-300	42.37 ± 5.23	48.49 ± 3.45	**0.0015**	43.02 ± 5.03	48.41 ± 3.133	**0.026**
CMT	295.8 ± 61.00	246.77 ± 21.30	**0.0131**	266.40 ± 33.78	255.17 ± 12.73	0.44
Parafoveal thickness	383.23 ± 57.91	313.92 ± 23.00	**0.001**	350.83 ± 22.34	325.83 ± 17.00	**0.034**
Overall thickness	368.46 ± 49.32	303.15 ± 20.07	**0.0007**	340.00 ± 20.95	317.33 ± 16.50	**0.041**

Compared by paired Student’s t-test. A *P* value < 0.05 is considered statistical significance and are shown in boldface

Then, we explored the correlation between BCVA and each parameter in patients with CRAO. As shown in Table 3, the Spearman correlation indicated that only CMT was significantly correlated with BCVA with a *P* value of 0.0244.

**Table 3:** Spearman correlation between BCVA (LogMAR) of CRAO patients and other parameters

	VD in SCP	VD in DCP	CMT	FAZ	AI	FD
r value	−0.244	−0.355	**0.626**	−0.383	−0.091	−0.457
*P* value	0.464	0.2378	**0.0244**	0.1715	0.7465	0.1350

R values and *P* values of the Spearmen correlation are shown in the table

A *P* value < 0.05 is considered statistical significance and are shown in boldface

**Fig. 3:**
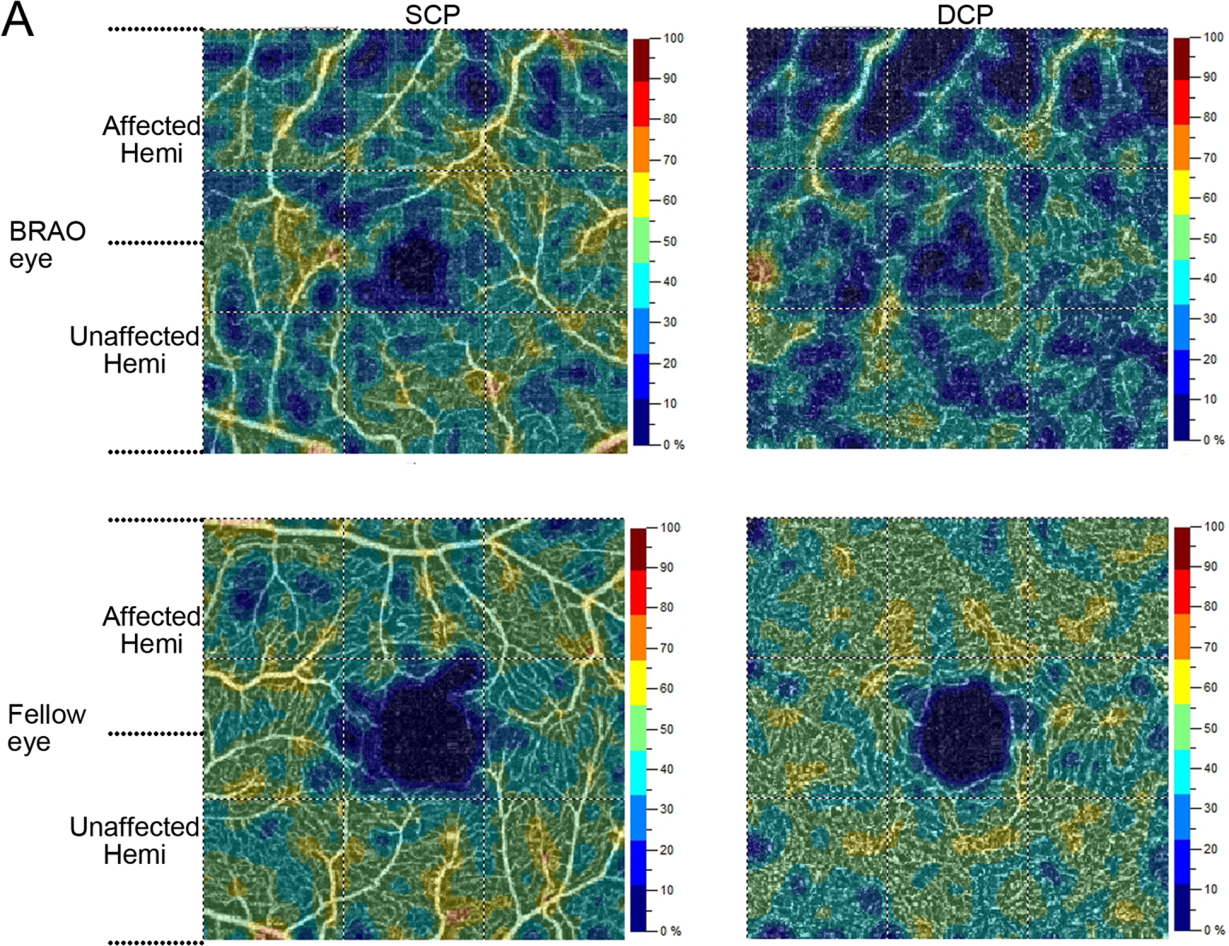
**a**:OCT-A image of a representative BRAO patient (upper temporal branch occlusion, the same patient in Additional file 1: Figure S1). Vessel density heat map of SCP, DCP of BRAO eye (superior) and the fellow eye (inferior)

In patients with BRAO, a significant reduction in vessel density in CC and FD-300 was observed (Table 2). The vessel density of the superior hemifield and inferior hemifield of the 3 × 3 macula area are shown separately. In BRAO patients, the vessel density of the RAO-affected hemifield was lower than that of the unaffected hemifield (Fig. 3, Table 4). Figure 3 is the OCT-A image of a patient with BRAO. The upper temporal branch of the retinal artery was occluded (as shown in FA in Additional file 1: Figure S1). The vessel density of the superior hemifield SCP and DCP was clearly lower than that of the inferior hemifield. More interestingly, the low-perfusion area did not appear to be limited to the RAO-affected hemifield; the vessel density of the unaffected hemifield of SCP and DCP also appeared to be reduced compared with that of fellow eyes (Fig. 3, Table 4), although no significance difference was observed (Table 4).

**Table 4:** Comparison of mean vessel density in different hemifield of the 6 BRAO eyes and unaffected fellow eyes

	SCP	DCP
Affected Hemifield (BRAO eyes, *n* = 6)	36.62 ± 6.40	40.42 ± 7.29
Unaffected Hemifield (BRAO eyes, *n* = 6)	39.10 ± 5.13	43.60 ± 5.94
*P* value	**0.048**	**0.0063**
Affected Hemifield (BRAO eyes, *n* = 6)	36.62 ± 6.40	40.42 ± 7.29
Affected Hemifield (Fellow eyes, *n* = 6)	42.88 ± 3.45	44.20 ± 2.85
P value	0.091	0.078
Unaffected Hemifield (BRAO eyes, *n* = 6)	39.10 ± 5.13	43.60 ± 5.94
Unaffected Hemifield (Fellow eyes, *n* = 6)	44.20 ± 2.85	45.95 ± 3.99
P value	0.124	0.201

Compared by paired Student t-test. A *P* value < 0.05 is considered statistical significance and are shown in boldface

**Fig. 4:**
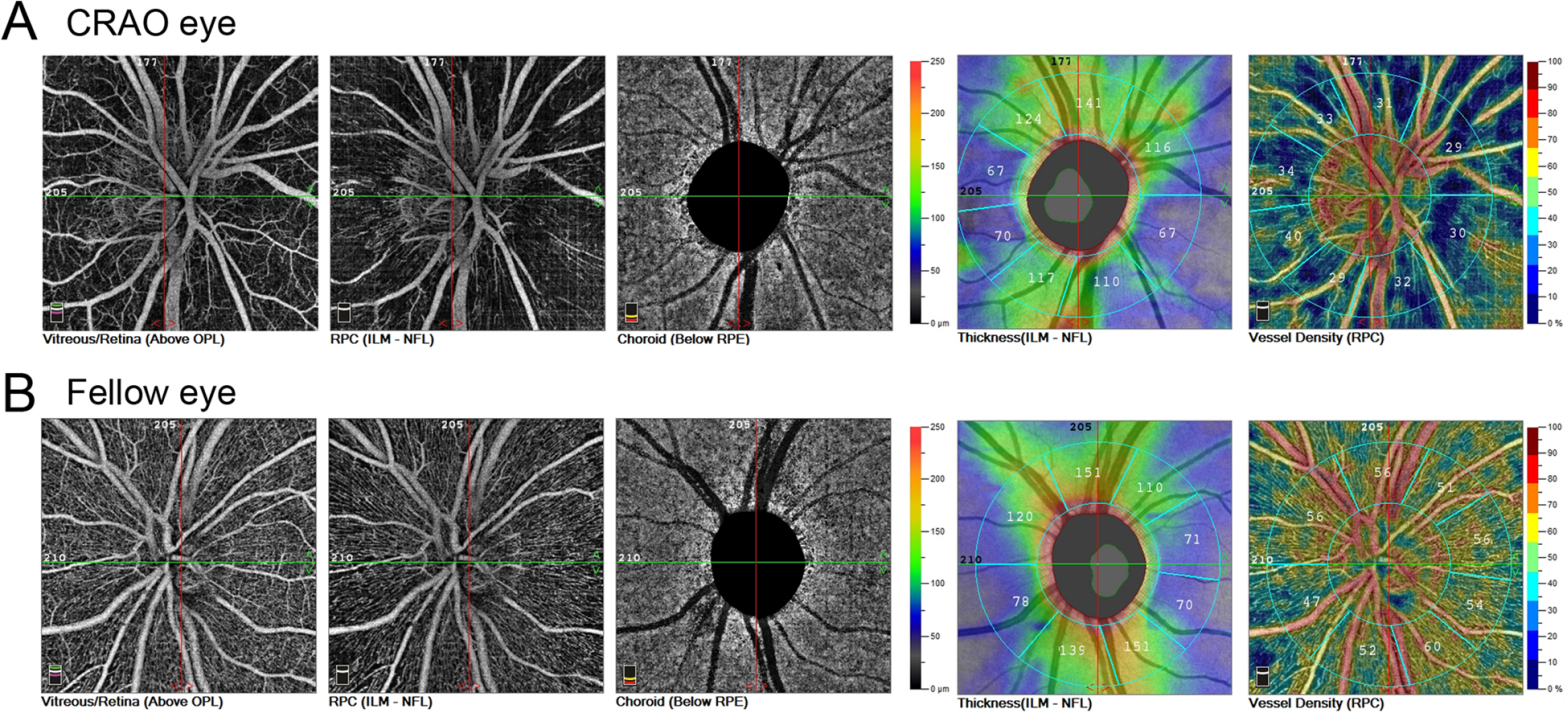
Optic disc OCT-A image of a CRAO patient. **a** CRAO eye; **b** fellow eye

We then investigated the vessel density around the optic disc and RNFL thickness of these patients. Since this study was retrospective, the archives of optic disc OCT-A images were available from only three CRAO patients. In all three patients, RPC density (whole image), peripapillary vessel density, and RNFL thickness were reduced compared those in fellow eyes (Table 5, Fig. 4). The inside disc vessel density was reduced in 2 of the 3 patients. In addition, OCT-A imaging clearly showed thinner (Fig. 4) peripapillary vessels.

**Table 5:** RPC densities and RNFL thickness in 3 CRAO patients

	RPC density (whole image)		Inside Disc		Peripapillary		RNFL Thickness (μm)	
	CRAO	Fellow	CRAO	Fellow	CRAO	Fellow	CRAO	Fellow
Patient 1	37.3	51.1	60.9	53.3	32.5	53.9	101	111
Patient 2	44.6	50.5	52.8	54.4	44.6	52.0	91	110
Patient 3	46.0	51.6	43.8	49.7	47.2	53.4	128	135

## Discussion

Bonini Filho M et al. previously reported that in most RAO patients, OCT-A revealed reduced vascular perfusion in both SCP and DCP qualitatively. In addition, the reduced vascular perfusion corresponded to the delayed dye perfusion areas in FA [4]. Here in our study, we revealed and quantified the OCT-A characteristics of acute RAO patients.

OCT-A is a novel method for imaging retinal vessels non-invasively and is capable of showing retinal vessels in different layers separately. The neural retina receives oxygen and nutrition from two independent circulation systems: the inner retina is supplied by the retinal artery system, while the outer retina receives oxygen and nutrition from the choroidal circulation system. Since RAO is a result of acute onset retinal arterial occlusion, the reduction in vessel densities in SCP, DCP, and FD-300, and the thickening of the inner retinal layer are understandable. Lavin P. et al. indicated that CRAO patients are at higher risk of future cardiovascular and cerebrovascular events [6]. Interestingly, we found that vessel density in SCP was reduced in RAO fellow eyes compared with that in normal control eyes. This result implies that in RAO patients, chronic microvascular change may exist before the onset of RAO. These microvascular changes may lead to the onset of RAO or even other cardio-cerebrovascular events. Thus, the reduced vessel density in SCP might act as a potential predictive factor for RAO. However, considering the retrospective nature of this study, our results should be treated with caution, and future prospective cohort studies are needed to confirm this finding.

Previous studies indicated that FAZ [7] is altered in some vascular-related diseases, such as like diabetic retinopathy [8, 9] and retinal vein occlusion (RVO) [10]. FAZ is also correlated with visual acuity in RVO [3] and diabetic retinopathy without diabetic macular edema [9]. However, in our current study, we found no significant alteration in FAZ in either CRAO or BRAO patients. In addition, no correlation was found between FAZ and visual acuity in CRAO patients.

Two additional parameters were recently derived from FAZ: AI and FD-300. AI is calculated as the perimeter of FAZ divided by the standard circular perimeter of an equal area [11–13]. AI is used to assess the circularity of FAZ. Recent studies have indicated that in diabetic retinopathy, AI is more sensitive than FAZ in revealing microvascular changes induced by the progression of diabetic retinopathy (DR) [11–13]. Consistently, our study also found increased AI in CRAO and BRAO eyes compared with that in fellow eyes. FD-300 represents the vessel density of the full retina around FAZ within a width of 300 μm. FD-300 was significantly decreased in DR compared with that in non-diabetic retinopathy controls and was negatively correlated with the stage of DR [2]. Here our study indicated that FD-300 is also decreased in CRAO and BRAO eyes compared with that in fellow eyes.

Our analysis of BRAO eyes addressed a reduction in vessel density in both SCP and DCP in the RAO-affected hemifield compared to that in the unaffected hemifield. Samara W et al. found that in BRVO patients, the vessel density of both SCP and DCP is lower in the RVO-affected hemifield than in the corresponding hemifield of the unaffected eye. Furthermore, the vessel density of DCP was reduced in the unaffected hemifield of the BRVO eye compared to that in the corresponding hemifield of the fellow eye (*P* = 0.04) [3]. In our study, though a trend was observed, after statistical analysis, we found no significant difference in the retinal vessel densities of the BRAO-affected hemifield compared with those of the corresponding hemifield of the fellow eye. We also found no significant difference in retinal vessel densities of the unaffected hemifield of the BRAO eye compared with those of the corresponding hemifield of the fellow eye. One explanation is that our BRAO sample size is relatively small.

Our results indicated that CMT is positively correlated with BCVA recorded by LogMAR; as macular edema becomes more severe, the visual acuity of the patient worsens. More severe macular edema indicated a higher degree of retinal ischemia, thus leading to worse BCVA. This result is consistent with the result of Ahn S et al. who recovered that the initial macular edema in CRAO patients was significantly correlated with final BCVA, though they did not record the initial BCVA [14].

The RPCs comprise straight, long vessels located within the RNFL that arise from peripapillary retinal arterioles. A previous study indicated an attenuation of RPC in RAO patients by OCT-A qualitatively [4]. Here we showed the quantified reduction in the vessel density of RPCs and parapapillary vasculatures in 3 CRAO patients. Yu P et al. demonstrated a correlation between RNFL thickness and RPC volume in normal human donor eyes [15]. These authors claimed that a positive correlation between RNFL thickness and RPC volume suggests a supportive role of RPCs for the RNFL. In our study, we also found that the reduced RPC vessel density was accompanied by a thinner RNFL thickness, at least in 3 patients. Thus, in CRAO patients, the reduced RPC vessel density due to ischemia might lead to the reduced thickness of the RNFL.

There are limitations to our study. First, since this study was retrospective, some of the results should be treated with caution, and future prospective studies are needed. Second, our sample size is relatively small, especially when analysing the optic-disk-centered images. In addition, OCT-A images can capture only a relatively small area around the macula or optic disc. Information outside captured area is missed.

## Conclusions

Our results indicated that OCT-A is capable of precisely quantifying the reduction in capillary plexus perfusion at both the superficial and deep layers in RAO patients. Novel parameters calculated from AngioVue Analytics addressed more characteristics in both RAO eyes and fellow eyes, thus, these parameters are promising for establishing more important clinical relevance for RAO. Therefore, OCT-A is a novel valuable tool for evaluating the ischemic changes in RAO patients.

## Additional file

Additional file 1: **Figure S1.** A. FA image of a BRAO patients. B. enlarged image indicated in A. C. OCT-A image center-ed on the fovea of the macula of the same patients. *represent the corresponding low-perfusion area in FA and OCT-A. (TIF 940 kb)

## Data Availability

The datasets used and/or analyzed during the current study available from the corresponding author on reasonable request.

## Abbreviations

AI: Acircularity Index
BCVA: Best-Corrected Visual Acuity
BRAO: Branch Retinal Artery Occlusion
CMT: Central Macular Thickness
CRAO: Central Retinal Artery Occlusion
DCP: Deep Capillary Plexus
DR: Diabetic Retinopathy
FA: Fluorescein Angiography
FAZ: Foveal Avascular Zone
OCT-A: Optical Coherence Tomography Angiography
RAO: Retinal Arterial Occlusion
RNFL: Retinal Nerve Fiber Layer
RPC: Radial Peripapillary Plexus
SCP: Superficial Capillary Plexus
SD: Standard Deviation
SSADA: Split Spectrum Amplitude Decorrelation Angiography
