# Supplementary figures and images for: Optical coherence tomography angiography characteristics of acute retinal arterial occlusion
Source: BMC Ophthalmol. 2019 Jul 10;19:147. doi: 10.1186/s12886-019-1152-8 (PMC6621973; doi:10.1186/s12886-019-1152-8)

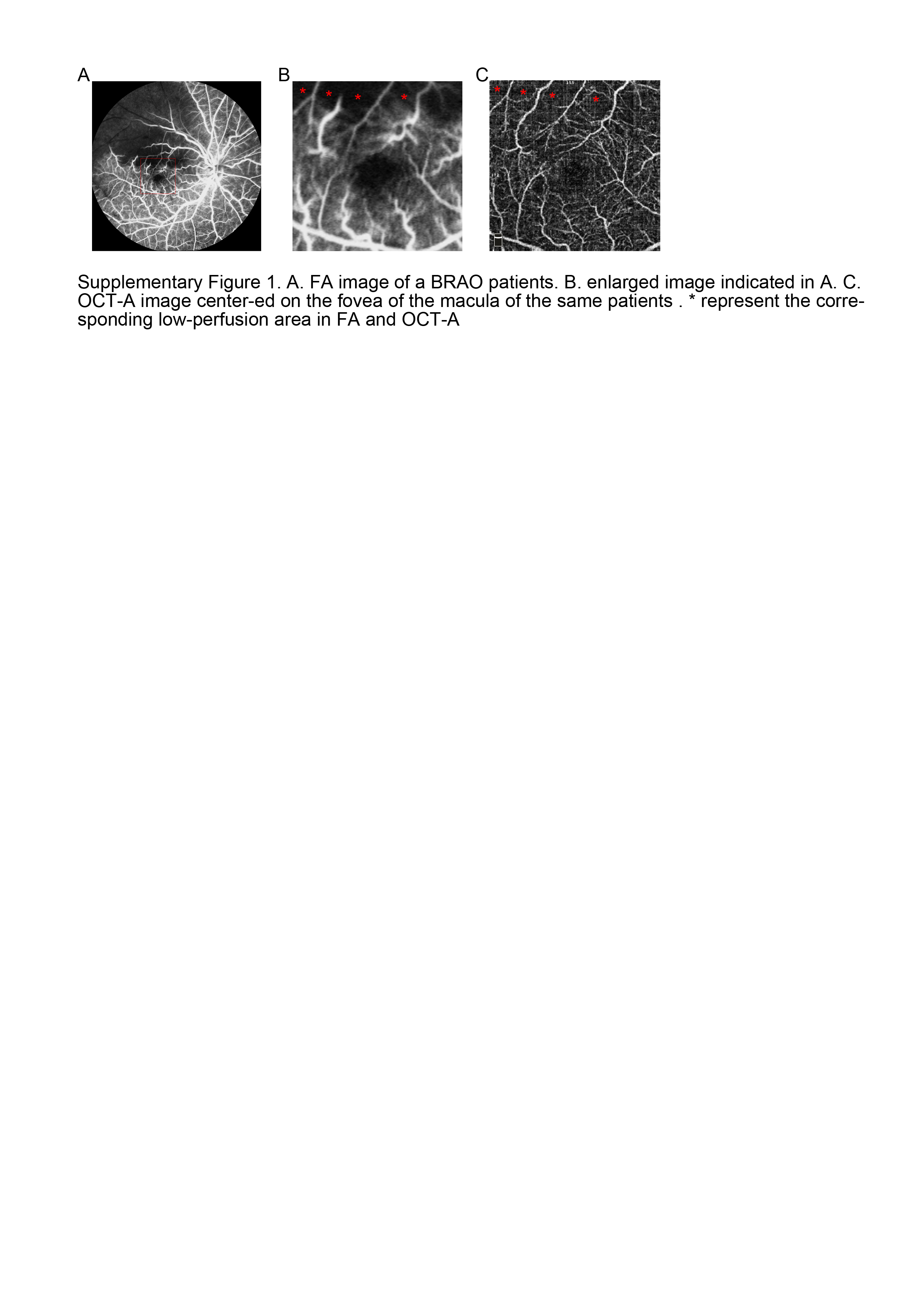

Supplement: Supplementary file 1 — Figure S1. A. FA image of a BRAO patients. B. enlarged image indicated in A. C. OCT-A image center-ed on the fovea of the macula of the same patients. *represent the corresponding low-perfusion area in FA and OCT-A. (TIF 940 kb) [file 12886_2019_1152_MOESM1_ESM.tif]
